# Chimerism, the Microenvironment and Control of Leukemia

**DOI:** 10.3389/fimmu.2021.652105

**Published:** 2021-04-22

**Authors:** H. Joachim Deeg

**Affiliations:** Fred Hutchinson Cancer Research Center and the University of Washington, Seattle, WA, United States

**Keywords:** chimerism, microenviroment, microbiome, GVHD prophylaxis regimens, allogeneic transplant, Graft vs. Leukemia Effect

## Abstract

Transplantation of allogeneic hematopoietic cells faces two barriers: failure of engraftment due to a host versus graft reaction, and the attack of donor cells against the patient, the graft versus host (GVH) reaction. This reaction may lead to GVH disease (GVHD), but in patients transplanted due to leukemia or other malignant disorders, this may also convey the benefit of a graft versus leukemia (GVL) effect. The interplay of transplant conditioning with donor and host cells and the environment in the patient is complex. The microbiome, particularly in the intestinal tract, profoundly affects these interactions, directly and via soluble mediators, which also reach other host organs. The microenvironment is further altered by the modifying effect of malignant cells on marrow niches, favoring the propagation of the malignant cells. The development of stable mixed donor/host chimerism has the potential of GVHD prevention without necessarily increasing the risk of relapse. There has been remarkable progress with novel conditioning regimens and selective T-cell manipulation aimed at securing engraftment while preventing GVHD without ablating the GVL effect. Interventions to alter the microenvironment and change the composition of the microbiome and its metabolic products may modify graft/host interactions, thereby further reducing GVHD, while enhancing the GVL effect. The result should be improved transplant outcome.

“… he first commanded Bellerophon to kill that savage monster, the Chimaera, who was not a human being, but a goddess, …” (Homer, *The Iliad*)

## Introduction

In modern medical terminology, particularly in transplantation, the term “chimera” is applied to the result of transplantation, specifically the transplantation of *cells* from one individual into another. This cell transfer will change the recipient composition (1) and may lead to adverse events by inducing a syndrome, termed graft versus host (GVH) disease (GVHD). While GVHD is undesirable, the transferred cells also aid in eliminating the disease for which the patient is being transplanted, via a graft versus leukemia (GVL) effect. In fact, conditioning with cytotoxic therapy alone generally will not eradicate the last malignant cells, as shown in early murine models (2). The donor cell-mediated GVL effect is an essential part of the curative potential of hematopoietic cell transplantation (HCT).

## Graft Versus Host Disease and Graft Versus Leukemia Effect

A GVL effect was first reported by Barnes and colleagues in murine models in 1956 (3) and 1957 (4), just as Don Thomas et al. reported the successful transfer of normal blood-forming hematopoietic stem cells from healthy donors into human patients with leukemia (5). These reports were followed by publications considering immunotherapeutic approaches to treat leukemia (6, 7). Weiden et al. presented the first comprehensive analysis of clinical transplant results, which showed that patients with acute leukemia who were transplanted with marrow cells from human leukocyte antigen (HLA)-matched sibling donors and who developed GVHD, particularly in its chronic form, had a reduced incidence of relapse and superior survival (8, 9). The GVH reaction is triggered by the encounter of cells from two individuals, the transplant donor and the recipient, with prominent manifestations at the patient's boundaries, in particular the intestinal tract (1, 10). Since a patient's leukemic cells have the same basic genetic makeup as the patient's healthy organs and tissues, this GVL effect may not be surprising. However, the question that arises immediately is whether this effect could be achieved and exploited without the development of GVHD. It has been challenging to separate the GVL effect from GVHD, but animal models indicate that the post-transplant interaction of donor and host cells—conventional and regulatory T cells, donor and host dendritic cells of various lineages, and iNKT cells, along with components of the microenvironment—can be shifted such that GVHD is largely prevented while the GVL effect is maintained (11).

The probability of post-HCT relapse depends upon numerous factors, including disease characteristics, treatment received before transplantation, remission status, including measurable residual disease (MRD) at the time of transplantation, the transplant conditioning regimen, the source of donor cells, HLA mismatch between donor and patient, and the development of (chronic) GVHD. MRD, in particular, is currently an area of extensive research. The level of detection of MRD depends upon the methodology used (e.g., deep sequencing for DNA mutations vs. multi-color flow cytometric analysis) (12–14). While flow cytometry identifies immunophenotypic abnormalities that may serve as targets for the GVH reaction and the GVL effect, this is less likely to be the case for most mutations, unless they result in changes in protein expression. A head-to-head comparison of flow and mutation data in regard to their impact on post-transplant relapse is currently not available. Further, there has been a keen interest in the role of DNA polymorphism (and the respective differences between donor and patient) and the occurrence of GVHD and GVL reactivity. While some single-nucleotide polymorphisms associated with a limited number of genes and their possible role for GVHD have been described, no firm conclusions can be drawn (15). Considering an impact of cytogenetic risk and GVHD, we carried out an analysis (Radich and Deeg, unpublished) in patients transplanted for MDS, selecting cohorts, which by conventional criteria could be considered the two extremes for relapse risk: patients who had high risk cytogenetics (16) and did not develop GVHD (acute or chronic) and patients with good risk karyotype who did develop GVHD. Remission status at the time of transplantation, donor selection, conditioning regimen, and GVHD prophylaxis were comparable. Contrary to our hypothesis that there would be a high incidence of relapse in the first cohort and a low incidence in the second, we failed to observe a significant difference. While the analysis may have had limited statistical power, the lack of any difference was striking. Clearly, risk parameters such as DNA mutations (17) (not available for our analysis) and factors that have not been incorporated into currently used risk schemes are relevant for relapse or sustained remission. It is of interest in this context that a recent report suggests a higher incidence of chronic GVHD and possibly a reduction in relapse incidence in patients transplanted from donors with clonal hematopoiesis (18).

## T-Cell Depletion

Early data on global T-cell depletion of the donor cell inoculum before infusion into the patient showed substantial reduction of the incidence of GVHD but also resulted in a high rate of graft failure and disease relapse (19). More recent data using selective T-cell depletion appear to be more promising.

One strategy is the administration of post-transplant cyclophosphamide (CY), originally for HLA haplo-identical transplants but then extended to other donor/host combinations (20). The reduction of the incidence of GVHD, especially chronic GVHD, with this approach was interpreted as a result of the elimination of host-alloreactive donor T cells. However, more recent data from murine models show that treatment with CY favors the development of CD4+CD25+Foxp3+ regulatory T cells. In addition, some conventional alloreactive T cells persist, albeit with impaired function (21). It is this conjunction of an expansion of regulatory T cells, including those with alloantigen specificity, and altered immuno-competence of conventional T cells that is responsible for the observed prevention of GVHD (21). This mechanism was also functional in thymectomized mice, indicating that it does not require the generation or central selection of T cells. Whether the use of post-transplant CY is associated with an increase in relapse, particularly of myeloid malignancies, remains a matter of debate. Apparently, the modified donor-derived alloreactive T cells maintain GVL activity.

Another concept with similar aims, the prevention of GVHD without increasing the risk of relapse, is the depletion of CD45A+CD62L+ naïve T cells (22, 23). In murine models, the infusion of naïve T cells induced severe GVHD, while central memory T cells resulted in milder GVHD, and effector-memory T cells did not cause significant GVHD (24, 25). Memory T cells, however, conveyed anti-pathogen immunity and GVL reactivity (26). Naïve CD45+CD62L+ T cells appear to be “uncommitted” and, thus, are able to get activated by new (patient) antigens that they encounter, thereby triggering a cascade of signals that initiate GVHD. In the clinic, patients with myeloid or lymphoid malignancies conditioned with regimens of various intensities and infused with hematopoietic cells from HLA-identical sibling donors that were *in vitro* depleted of CD45RA+ T cells achieved sustained engraftment, had a very low incidence of severe acute and chronic GVHD, and were not at a higher risk of relapse than patients transplanted with T cell-replete grafts (23). Further, in patients who did develop acute GVHD, generally grade II, corticosteroid treatment could be discontinued much earlier, at a median of 85 days, compared with 853 days in patients given T cell-replete grafts. No case of steroid-refractory GVHD has been observed so far after naïve T-cell depletion. This pattern of rapid response of acute GVHD to steroid therapy and the rare occurrence of chronic GVHD suggests a modified immune environment and a different biology of acute GVHD related to the removal of non-committed naïve T cells. The fact that regulatory T cells that express CD45RA are also eliminated suggests that those cells are not required for the establishment or maintenance of tolerance in this clinical model. In fact, one can speculate that elimination of those regulatory T cells might lead to a more potent GVL effect.

## Mixed Donor/Recipient Chimerism

What is the impact of *incomplete* donor cell engraftment? Available data indicate that the development of *mixed* chimerism, the concurrent presence of recipient and donor lympho-hematopoietic cells in the patient after transplantation, may attenuate or prevent the development of GVHD. Mixed chimerism was originally described in patients with *non-malignant disorders*, in particular immune deficiencies (27) but also in aplastic anemia (28). This mixed chimerism can persist for years. Studies in a canine model indicated that administration of sublethal doses of total body irradiation before and pharmacological immunosuppression after donor cell infusion resulted in stable mixed hematopoietic donor/recipient chimerism (29). These data underscore the importance of the intensity of the transplant conditioning regimens, which for non-malignant disorders tend to be less intensive, for the development of mixed chimerism.

Would mixed chimerism also be possible and consistent with transplant success in patients with *malignant disorders*? Stated differently, would the establishment of “tolerance” between patient and recipient cells include tolerance to the malignant cells and, thereby, eliminated the GVL effect? In fact, several reports have shown persistent antitumor responses *even after a loss of donor cell chimerism* (30, 31). What is the mechanism? The answer will at least in part depend upon which donor and patient cell sub-populations in the patient's marrow and immune system account for the mix and how the mix alters cell functions. We showed recently that in patients transplanted for myeloproliferative disorders, mixed CD33+ chimerism was associated with subsequent relapse, whereas mixed CD3+ chimerism was not and, in fact, did result in less GVHD without an increased incidence of relapse (32). We observed similar outcomes in two trials enrolling patients with acute myeloid leukemia (AML) or MDS who had been conditioned with busulfan/fludarabine and thymoglobulin (Yeh et al., unpublished observations, February 2021). The factors controlling this balance between patient and donor cells without leading to disease recurrence remain to be determined.

## Graft Versus Leukemia Effects Without Classical Hematopoietic Cell Transplantation

If cells from healthy donors are able to induce a GVL effect after transplantation, can such an effect be achieved with the infusion of donor cells (DLI), *without carrying out an actual transplant*, as has been shown for patients who relapsed after transplantation (60)? Several investigators used leukocyte infusions from HLA-mismatched donors in an attempt to provide a direct GVL effect (33–35). In one study, DLI was given to patients with various malignancies to induce a GVL or GV tumor effect (33). These patients were pre-treated with interferon 2β and given DLI, and 4 weeks later, donor chimerism (determined by PCR for marker analysis) was detected in four of 11 evaluable patients. Of note, four patients who had previously received an autologous transplant developed acute GVHD, and the three patients who could be assessed did show anti-tumor responses. GVHD is a risk associated with DLI. However, the occurrence of GVHD in patients who had previously undergone a transplant is consistent with a modified microenvironment and a role of host cells in the GVHD pathophysiology (36). However, many patients given DLI for relapse after transplantation do experience tumor responses *without developing GVHD*, illustrating that *clinical* GVHD is not required for a GVL effect to occur. The GVL effect may be mediated by a subclinical reaction or, alternatively, might involve activity against antigens with limited expression, restricted to the tumor (37). Ongoing research is exploiting this possibility, for example, by generating effector cells against minor histocompatibility antigens (HA-1) primarily expressed on lympho-hematopoietic cells and for which patient and donor differ (61).

Guo et al. reported results with the infusion of granulocyte colony-stimulating factor (G-CSF)-mobilized peripheral blood progenitor cells (including CD34+, CD3+, and NK cells) at various doses, from HLA-mismatched donors in patients with AML in first remission who were not given any GVHD prophylaxis (38). Donor chimerism as determined by the identification of cells containing the Y chromosome (from the male donor) was present in 20 of 23 female recipients as late as 1,000 days after infusion. Leukemia-free survival was significantly prolonged in patients who received higher doses of CD3+ donor cells, while no GVHD was observed. The investigators also showed, further, that in addition to a *GVL* effect, these patients also experienced a *recipient vs. leukemia* effect, suggesting activation of the patient's immune system by the infused HLA non-identical donor cells (38). The same authors subsequently reported similar results in another 185 patients with *de novo* AML (35), but confirmation from other centers is currently not available.

## The Microenvironment

The marrow microenvironment is essential for the support of normal and malignant hematopoiesis. We have presented *in vitro* data from patients with MDS, which show a two-way signaling path between the clonal disease cells and non-clonal mesenchymal/stroma cells (39). Stroma cells exhibited altered gene expression and favored the survival of clonal MDS cells rather than healthy hematopoietic precursors. Exposure to the hypomethylating agent, 5-aza-citidine, normalized gene expression in stroma cells and restored their functional competence in support of normal hematopoiesis (39). It is intriguing to speculate that altered gene expression in the marrow microenvironment is a contributor to the frequently observed myelosuppression following DLI.

Data on the role of stroma in disease persistence or recurrence have also been presented for patients with AML (40, 41). Those studies show that malignant (clonal) myeloid cells trigger remodeling events in bone marrow niches, and this remodeled environment then favors the expansion of the malignant clone (39, 42). Other broad-acting contributors to the altered post-transplant milieu in the patient are the effects of endothelial cell activation (43).

Further, solid cancer models show that propagation of clonal tumor cells in the form of metastases was dependent upon the co-migration of stromal cells with those tumor cells (44). Consistent with that observation, we were not able to establish sustained engraftment of clonal MDS cells in a xenotransplant model of human MDS cells in immunodeficient mice, if MDS cells were injected by themselves. However, we did achieve long-term engraftment and expansion when MDS cells were injected along with the (transformed) human stroma cell line HS27a (45). The role of the microenvironment for effective hematopoiesis is undisputed, but what is of note in these models is the support of the *clonal disease* that is mediated at least in part by a quasi auto-feedback loop that leads to “preferential treatment” of the clone.

Possibly related to these data are observations on the development of donor-derived leukemia, i.e., the transformation of polyclonal, healthy donor cells into originator cells of a clonal myeloid disorder (assuming the absence of preexisting clonal abnormalities in donor cells). Several reports have postulated a “leukemogenic effect” of the marrow microenvironment (46, 47). Is the underlying mechanism related to signals provided by donor cells, viz., the chimeric status associated with a successful transplant?

## The Microbiome

Exciting research has established that the microbiome plays a central role in the development of GVHD (48–50). We recently summarized data from several laboratories on the profound effects of donor/host interactions at the boundaries of the transplant recipient and the role of the patient's microbiome, particularly in the intestinal tract, in modifying those interactions (1). Shifts in the composition of the intestinal microbiome are associated with GVHD. While some bacteria, such as *Blautia*, appear to have a beneficial effect, others, for example, *Veillonella* or enterococcal species such as *Enterococcus faecium* or *Enterococcus faecalis*, favor the development or propagation of GVHD, leading to inferior transplant survival (51). These intestinal bacteria interact directly with patient cells, including GALT, L cells, and dendritic cells and thereby modify either tolerogenic or allo-reactive signals (52, 53). Various species, such as *E. faecalis*, can cross the intestinal barrier and migrate to intestinal lymph nodes, priming resident T and B lymphocytes. Bacterial metabolites, specifically the short-chain fatty acids butyrate or propionate, released into the bloodstream, have a protective effect against chronic GVHD (54, 55). One mechanism involves enhanced development of regulatory T cells. Conversely, a loss of species that produce high levels of butyrate would be associated with a higher incidence of GVHD. So far, there is no evidence that a shift in the composition of the intestinal microbiome impacted progression of the malignancy for which the patient underwent transplantation (51), although there is a profound impact of the mix of the gut microbiome on the response to immunotherapy in other models (56). Intriguing are some very recent observations (Chris Johnston PhD, personal communication, November 2020) indicating that bacteria can alter the methylation pattern of human DNA, thereby modifying gene expression. Conceivably, this may lead to alterations of potential targets for a GVL effect by donor cells.

Viral organisms such as picobirna viruses have also been shown to participate in these donor/host interactions (57), and the role of the cytomegalovirus (CMV) in GVHD development has been investigated extensively (58). Sellar et al. (59) studied patients with various lymphohematopoietic malignancies who were CMV+ and received transplants from CMV negative donors. The conditioning regimens were of reduced intensity and included *in vivo* T-cell depletion with alemtuzumab. The investigators showed that CMV-specific T cells were exclusively of *host* origin and protected the patients against recurrent CMV infections, indicating that the status of mixed donor/host chimerism in these patients was associated with increased immune protection. DLI to induced full donor chimerism did not trigger the development of symptomatic CMV infection, and in some patients, *donor-derived* CMV-specific CD8+ T lymphocytes further expanded. This conversion (from host to donor) occurred without clinical evidence of GVHD, suggesting the possibility that the presence of mixed chimerism, albeit temporary, facilitated the establishment of tolerance.

## Summary and Conclusions

The interactions between donor and recipient cells following allogeneic HCT are complex, and the cast of characters of this drama is not limited to donor and recipient immune cells. Additional actors include cellular and non-cellular components of the microenvironment and, importantly, the microbiome. Nature had not envisioned *Homo sapiens* trying to break down barriers that have evolved over millions of years. Doing so upsets the balance that we observe in healthy individuals. Of course, these therapeutic interventions are directed at the eradication of a malignant disease, which has already changed the internal milieu. A better understanding of signals that trigger the development of malignant disorders such as leukemia would allow for earlier interventions and might permit their exploitation to restrict the reactions of donor cells to the GVL effect, while preventing GVHD. Can we direct the divine ability of the chimera against the malignancy and sever the ugly head of GVHD? Current research using state of the art tools, including systems biology and machine learning, may be able to pave the way.

## Data Availability Statement

The original contributions presented in the study are included in the article/supplementary material, further inquiries can be directed to the corresponding author/s.

## Author Contributions

The author confirms being the sole contributor of this work and has approved it for publication.

## Conflict of Interest

The author declares that the research was conducted in the absence of any commercial or financial relationships that could be construed as a potential conflict of interest.

## References

[1] Deeg HJ. Individuals, boundaries, graft-versus-host disease. Biol Blood Marrow Transplant. (2020) 26:e309–12. 10.1016/j.bbmt.2020.09.001 32927076

[2] Burchenal JH Oettgen HF Holmberg EA Hemphill SC Reppert JA. Effect of total body irradiation on the transplantability of mouse leukemias. Cancer Res. (1960) 20:425 13806002

[3] Barnes DWH Corp MJ Loutit JF Neal FE. Treatment of murine leukaemia with x-rays and homologous bone marrow. preliminary communication. Br Med J. (1956) 2:626–7. 10.1136/bmj.2.4993.626 13356034 PMC2035298

[4] Barnes DWH Loutit JF. Treatment of murine leukaemia with x-rays and homologous bone marrow: II. Br J Haematol. (1957) 3:241–52. 10.1111/j.1365-2141.1957.tb05793.x 13460193

[5] Thomas ED Lochte HL Jr Lu WC Ferrebee JW. Intravenous infusion of bone marrow in patients receiving radiation and chemotherapy. N Engl J Med. (1957) 257:491–6. 10.1056/NEJM195709122571102 13464965

[6] Mathe G Amiel JL Schwarzenberg L Cattan A Schneider M. Adoptive immunotherapy of acute leukemia: experimental and clinical results. Cancer Res. (1965) 25:1525–31. 5323965

[7] Mathe G Amiel JL Schwarzenberg L Cattan A Schneider M Devries MJ. Successful allogeneic bone marrow transplantation in man: chimerism, induced specific tolerance and possible anti-leukemia effects. Blood. (1965) 25:179–96. 10.1182/blood.V25.2.179.179 14267694

[8] Weiden PL Flournoy N Thomas ED Prentice R Fefer A Buckner CD. Antileukemic effect of graft-versus-host disease in human recipients of allogeneic-marrow grafts. N Engl J Med. (1979) 300:1068–73. 10.1056/NEJM197905103001902 34792

[9] Weiden PL Sullivan KM Flournoy N Storb R Thomas ED the Seattle Marrow Transplant T. Antileukemic effect of chronic graft-versus-host disease. contribution to improved survival after allogeneic marrow transplantation. N Engl J Med. (1981) 304:1529–33. 10.1056/NEJM198106183042507 7015133

[10] Hill GR Ferrara JL. The primacy of the gastrointestinal tract as a target organ of acute graft-versus-host disease: rationale for the use of cytokine shields in allogeneic bone marrow transplantation. Blood. (2000) 95:2754–9. 10.1182/blood.V95.9.2754.009k25_2754_2759 10779417

[11] Morris ES Macdonald KP Hill GR. Stem cell mobilization with G-CSF analogs: a rational approach to separate GVHD and GVL? Blood. (2006) 107:3430–5. 10.1182/blood-2005-10-4299 16380448

[12] Schuurhuis GJ Heuser M Freeman S Bene MC Buccisano F Cloos J. Minimal/measurable residual disease in AML: a consensus document from the European LeukemiaNet MRD Working Party. Blood. (2018) 131:1275–91. 10.1182/blood-2017-09-801498 29330221 PMC5865231

[13] Hourigan CS Dillon LW Gui G Logan BR Fei M Ghannam J. Impact of conditioning intensity of allogeneic transplantation for acute myeloid leukemia with genomic evidence of residual disease. J Clin Oncol. (2020) 38:1273–83. 10.1200/JCO.19.03011 31860405 PMC7164487

[14] Dillon LW Gui G Logan BR Fei M Ghannam J Li Y. Impact of conditioning intensity and genomics on relapse after allogeneic transplantation for patients with myelodysplastic syndrome. JCO Precis Oncol. (2021) 5:265–74. 10.1200/PO.20.00355 PMC8140814 34036237

[15] Martin PJ Fan W Storer BE Levine DM Zhao LP Warren EH. Replication of associations between genetic polymorphisms and chronic graft-versus-host disease. Blood. (2016) 128:2450–6. 10.1182/blood-2016-07-728063 27758874 PMC5114491

[16] Schanz J Steidl C Fonatsch C Pfeilstocker M Nosslinger T Tuechler H. Coalesced multicentric analysis of 2,351 patients with myelodysplastic syndromes indicates an underestimation of poor-risk cytogenetics of myelodysplastic syndromes in the international prognostic scoring system. J Clin Oncol. (2011) 29:1963–70. 10.1200/JCO.2010.28.3978 21519021 PMC4874202

[17] Bejar R. Implications of molecular genetic diversity in myelodysplastic syndromes. Curr Opin Hematol. (2017) 24:73–8. 10.1097/MOH.0000000000000313 27875374 PMC5433846

[18] Frick M Chan W Arends CM Hablesreiter R Halik A Heuser M. Role of donor clonal hematopoiesis in allogeneic hematopoietic stem-cell transplantation. J Clin Oncol. (2019) 37:375–85. 10.1200/JCO.2018.79.2184 30403573

[19] Kernan NA Flomenberg N Dupont B O'reilly RJ. Graft rejection in recipients of T-cell-depleted HLA-nonidentical marrow transplants for leukemia. identification of host-derived antidonor allocytotoxic T lymphocytes. Transplantation. (1987) 43:842–7. 10.1097/00007890-198743060-00014 3296349

[20] Fuchs EJ Luznik L. HLA-haploidentical hematopoietic cell transplantation. UpToDate [Electronic Resource] (2019). Available online at: https://www.uptodate.com/contents/hla-haploidentical-hematopoietic-cell-transplantation

[21] Wachsmuth LP Patterson MT Eckhaus MA Venzon DJ Gress RE Kanakry CG. Post-transplantation cyclophosphamide prevents graft-versus-host disease by inducing alloreactive T cell dysfunction and suppression. J Clin Invest. (2019) 129:2357–73. 10.1172/JCI124218 30913039 PMC6546453

[22] Bleakley M Heimfeld S Jones LA Turtle C Krause D Riddell SR. Engineering human peripheral blood stem cell grafts that are depleted of naive T cells and retain functional pathogen-specific memory T cells. Biol Blood Marrow Transp. (2014) 20:705–16. 10.1016/j.bbmt.2014.01.032 24525279 PMC3985542

[23] Bleakley M Heimfeld S Loeb KR Jones LA Chaney C Seropian S. Outcomes of acute leukemia patients transplanted with naive T cell-depleted stem cell grafts. J Clin Invest. (2015) 125:2677–89. 10.1172/JCI81229 26053664 PMC4563691

[24] Dutt S Tseng D Ermann J George TI Liu YP Davis CR. Naive and memory T cells induce different types of graft-versus-host disease. J Immunol. (2007) 179:6547–54. 10.4049/jimmunol.179.10.6547 17982043

[25] Zheng H Matte-Martone C Jain D Mcniff J Shlomchik WD. Central memory CD8+ T cells induce graft-versus-host disease and mediate graft-versus-leukemia. J Immunol. (2009) 182:5938–48. 10.4049/jimmunol.0802212 19414745 PMC9844260

[26] Zheng H Matte-Martone C Li H Anderson BE Venketesan S Sheng TH. Effector memory CD4+ T cells mediate graft-versus-leukemia without inducing graft-versus-host disease. Blood. (2008) 111:2476–84. 10.1182/blood-2007-08-109678 18045967 PMC2234071

[27] Heimall J Logan BR Cowan MJ Notarangelo LD Griffith LM Puck JM. Immune reconstitution and survival of 100 SCID patients post-hematopoietic cell transplant: a PIDTC natural history study. Blood. (2017) 130:2718–27. 10.1182/blood-2017-05-781849 29021228 PMC5746165

[28] Spitzer TR Himoe E Cottler-Fox M Cahill R Deeg HJ. Long-term stable mixed chimaerism following allogeneic marrow transplantation for severe aplastic anaemia. Br J Haematol. (1990) 76:146–54. 10.1111/j.1365-2141.1990.tb07850.x 2223634

[29] Storb R Yu C Wagner JL Deeg HJ Nash RA Kiem HP. Stable mixed hematopoietic chimerism in DLA-identical littermate dogs given sublethal total body irradiation before and pharmacological immunosuppression after marrow transplantation. Blood. (1997) 89:3048–54. 10.1182/blood.V89.8.3048 9108426

[30] Rubio MT Kim YM Sachs T Mapara M Zhao G Sykes M. Antitumor effect of donor marrow graft rejection induced by recipient leukocyte infusions in mixed chimeras prepared with nonmyeloablative conditioning: critical role for recipient-derived IFN-gamma. Blood. (2003) 102:2300–7. 10.1182/blood-2002-12-3949 12791660

[31] Dey BR Mcafee S Colby C Cieply K Caron M Saidman S. Anti-tumour response despite loss of donor chimaerism in patients treated with non-myeloablative conditioning and allogeneic stem cell transplantation. Br J Haematol. (2005) 128:351–9. 10.1111/j.1365-2141.2004.05328.x 15667537

[32] Deeg HJ Salit RB Monahan T Schoch G Mcfarland C Scott BL. Early mixed lymphoid donor/host chimerism is associated with improved transplant outcome in patients with primary or secondary myelofibrosis. Biol Blood Marrow Transplant. (2020) 26:2197–203. 10.1016/j.bbmt.2020.07.013 32693211 PMC8816377

[33] Porter DL Connors JM Van DV Duffy KM Mcgarigle C Saidman SL. Graft-versus-tumor induction with donor leukocyte infusions as primary therapy for patients with malignancies. J Clin Oncol. (1999) 17:1234. 10.1200/JCO.1999.17.4.1234 10561184

[34] Dickinson AM Norden J Li S Hromadnikova I Schmid C Schmetzer H. Graft-versus-leukemia effect following hematopoietic stem cell transplantation for leukemia. Front Immunol. (2017) 8:496. 10.3389/fimmu.2017.00496 28638379 PMC5461268

[35] Guo M Chao NJ Li JY Rizzieri DA Sun QY Mohrbacher A. HLA-mismatched microtransplant in older patients newly diagnosed with acute myeloid leukemia: results from the microtransplantation interest group. JAMA Oncol. (2018) 4:54–62. 10.1001/jamaoncol.2017.2656 28910431 PMC5833633

[36] Shlomchik WD. Graft-versus-host disease (Review). Nat Rev Immunol. (2007) 7:340–52. 10.1038/nri2000 17438575

[37] Kolb HJ. Graft-versus-leukemia effects of transplantation and donor lymphocytes (Review). Blood. (2008) 112:4371–83. 10.1182/blood-2008-03-077974 19029455

[38] Guo M Hu KX Liu GX Yu CL Qiao JH Sun QY. HLA-mismatched stem-cell microtransplantation as postremission therapy for acute myeloid leukemia: long-term follow-up. J Clin Oncol. (2012) 30:4084–90. 10.1200/JCO.2012.42.0281 23045576

[39] Bhagat TD Chen S Bartenstein M Barlowe AT Von Ahrens D Choudhary GS. Epigenetically aberrant stroma in MDS propagates disease via Wnt/beta-catenin activation. Cancer Res. (2017) 77:4846–57. 10.1158/0008-5472.CAN-17-0282 28684528 PMC5600853

[40] Konopleva M Konoplev S Hu W Zaritskey AY Afanasiev BV Andreeff M. Stromal cells prevent apoptosis of AML cells by up-regulation of anti-apoptotic proteins. Leukemia. (2002) 16:1713–24. 10.1038/sj.leu.2402608 12200686

[41] Tabe Y Konopleva M. Role of microenvironment in resistance to therapy in AML. Curr Hematol Malig Rep. (2015) 10:96–103. 10.1007/s11899-015-0253-6 25921386 PMC4447522

[42] Chen Y Hoffmeister LM Zaun Y Arnold L Schmid KW Giebel B. Acute myeloid leukemia-induced remodeling of the human bone marrow niche predicts clinical outcome. Blood Adv. (2020) 4:5257–68. 10.1182/bloodadvances.2020001808 33108453 PMC7594397

[43] Pagliuca S Michonneau D Sicre De Fontbrune F Sutra Del Galy A Xhaard A Robin M. Allogeneic reactivity-mediated endothelial cell complications after HSCT: a plea for consensual definitions. Blood Adv. (2019) 3:2424–35. 10.1182/bloodadvances.2019000143 31409584 PMC6693000

[44] Duda DG Duyverman AM Kohno M Snuderl M Steller EJ Fukumura D. Malignant cells facilitate lung metastasis by bringing their own soil. Proc Natl Acad Sci USA. (2010) 107:21677–82. 10.1073/pnas.1016234107 21098274 PMC3003109

[45] Li X Marcondes AM Ragoczy T Telling A Deeg HJ. Effect of intravenous coadministration of human stroma cell lines on engraftment of long-term repopulating clonal myelodysplastic syndrome cells in immunodeficient mice. Blood Cancer J. (2013) 3:e113. 10.1038/bcj.2013.11 23624784 PMC3641319

[46] Nafa K Bessler M Deeg HJ Luzzatto L. New somatic mutation in the PIG-A gene emerges at relapse of paroxysmal nocturnal hemoglobinuria. Blood. (1998) 92:3422–7. 10.1182/blood.V92.9.3422 9787183

[47] Aldoss I Song JY Curtin PT Forman SJ. Multiple donor-derived leukemias in a recipient of allogeneic hematopoietic cell transplantation for myeloid malignancy. Blood Adv. (2020) 4:4798–801. 10.1182/bloodadvances.2020002803 33022063 PMC7556138

[48] Penack O Holler E Van Den Brink MR. Graft-versus-host disease: regulation by microbe-associated molecules and innate immune receptors (Review). Blood. (2010) 115:1865–72. 10.1182/blood-2009-09-242784 20042727

[49] Jenq RR Taur Y Devlin SM Ponce DM Goldberg JD Ahr KF. Intestinal blautia is associated with reduced death from graft-versus-host disease. Biol Blood Marrow Transplant. (2015) 21:1373–83. 10.1016/j.bbmt.2015.04.016 25977230 PMC4516127

[50] Haak BW Littmann ER Chaubard JL Pickard AJ Fontana E Adhi F. Impact of gut colonization with butyrate-producing microbiota on respiratory viral infection following allo-HCT. Blood. (2018) 131:2978–86. 10.1182/blood-2018-01-828996 29674425 PMC6024637

[51] Stein-Thoeringer CK Nichols KB Lazrak A Docampo MD Slingerland AE Slingerland JB. Lactose drives Enterococcus expansion to promote graft-versus-host disease. Science. (2019) 366:1143–9. 10.1126/science.aax3760 31780560 PMC7003985

[52] Rescigno M Urbano M Valzasina B Francolini M Rotta G Bonasio R. Dendritic cells express tight junction proteins and penetrate gut epithelial monolayers to sample bacteria. Nat Immunol. (2001) 2:361–7. 10.1038/86373 11276208

[53] Chistiakov DA Bobryshev YV Kozarov E Sobenin IA Orekhov AN. Intestinal mucosal tolerance and impact of gut microbiota to mucosal tolerance. Front Microbiol. (2014) 5:781. 10.3389/fmicb.2014.00781 25628617 PMC4292724

[54] Zitvogel L Kroemer G. Immunostimulatory gut bacteria. Science. (2019) 366:1077–1078. 10.1126/science.aaz7595 31780546

[55] Markey KA Schluter J Gomes ALC Littmann ER Pickard AJ Taylor BP. The microbe-derived short-chain fatty acids butyrate and propionate are associated with protection from chronic GVHD. Blood. (2020) 136:130–6. 10.1182/blood.2019003369 32430495 PMC7332893

[56] Gopalakrishnan V Helmink BA Spencer CN Reuben A Wargo JA. The influence of the gut microbiome on cancer, immunity, cancer immunotherapy. Cancer Cell. (2018) 33:570–80. 10.1016/j.ccell.2018.03.015 29634945 PMC6529202

[57] Legoff J Resche-Rigon M Bouquet J Robin M Naccache SN Mercier-Delarue S. The eukaryotic gut virome in hematopoietic stem cell transplantation: new clues in enteric graft-versus-host disease. Nat Med. (2017) 23:1080–5. 10.1038/nm.4380 28759053

[58] Nichols WG Corey L Gooley T Davis C Boeckh M. High risk of death due to bacterial and fungal infection among cytomegalovirus (CMV)-seronegative recipients of stem cell transplants from seropositive donors: evidence for indirect effects of primary CMV infection. J Infect Dis. (2002) 185:273–82. 10.1086/338624 11807708

[59] Sellar RS Vargas FA Henry JY Verfuerth S Charrot S Beaton B. CMV promotes recipient T-cell immunity following reduced-intensity T-cell-depleted HSCT, significantly modulating chimerism status. Blood. (2015) 125:731–9. 10.1182/blood-2014-07-589150 25499763

[60] Kolb HJ Mittermüller J Clemm C Holler G Ledderose G Brehm G. Donor leukocyte transfusions for treatment of recurrent chronic myelogenous leukemia in marrow transplant patients. Blood. (1990) 76:2462–2465. 10.1182/blood.V76.12.2462.2462 2265242

[61] Krakow EF Summers C Dahlberg A Bar M Biernacki MA Cunningham T. Phase I study of adoptive immunotherapy with HA-1-specific CD8+ and CD4+ memory T cells for children and adults with relapsed acute leukemia after allogeneic Hematopoietic Stem Cell Transplantation (HCT): trial in progress. Blood. (2020) 136:45–6. 10.1182/blood-2020-137726

